# Narrative and active video game in separate and additive effects of physical activity and cognitive function among young adults

**DOI:** 10.1038/s41598-018-29274-0

**Published:** 2018-07-20

**Authors:** Jungyun Hwang, Amy Shirong Lu

**Affiliations:** Health Technology Lab, College of Arts, Media and Design, Bouvé College of Health Sciences, Northeastern University, Boston, MA 02115 USA

## Abstract

Physically active video games (AVGs) have been found to positively impact physical activity behaviors, especially when a narrative is added. However, the motivational and cognitive benefits of adding narrative to AVG are unclear. We examined the separate and additive effects of narrative and AVG on physical activity and cognitive function versus an active comparator, such as a sedentary video game (SVG). We randomly assigned young adults to one of four groups (narrative-AVG, AVG, narrative-SVG, or SVG) and had them complete sustained attention and working memory tasks before and after a 30-min experimental condition. Participants in both narrative-AVG and AVG groups achieved a moderate-intensity physical activity, while adding narrative to AVG resulted in higher step counts and more time spent in moderate-to-vigorous physical activity than AVG without narrative. Regardless of the narrative effect, participants in both AVG groups performed better on overall working memory than both SVG groups, while both AVG and SVG groups similarly achieved maximal performance in sustained attention. Working memory enhancement was positively correlated with increased heart rate. Participants in narrative-SVG group had a better response accuracy in working memory than those who played SVG without narrative. Taken together, adding narrative to AVG as a motivational component increased physical activity, which was the primary factor in the improvement of overall working memory.

## Introduction

Video gaming has become an increasingly popular form of entertainment and a part of modern popular culture worldwide in the last four decades. In 2017, 67% of United States (U.S.) households owned a device capable of playing a sedentary video game (SVG, a form of sedentary behavior)^1^ and at least one person in each household plays 3 hours or more of video games per week^2^. Young adults (aged 18 to 35 years old) represent a significant portion (27%) of the game-playing population. While playing an SVG may have some cognitive, motivational, emotional, and social benefits in clinical and non-clinical settings^3^, a growing body of evidence demonstrates lengthy sedentary game play’s negative impacts on health. These include health risks associated with increases in depression, aggressive behaviors, video game addiction, sleep deprivation, limited social or mental leisure activities^4^, and greater levels of obesity^5,6^ and musculoskeletal pain^7^.

Replacing SVGs with physically active video games (AVG, also known as exergaming), or “interactive video or electronic games that feature player movement, such as would occur in ‘real-life’ exercise participation”^8^, may be an alternate way to enhance overall health^9^. Some recent studies have indicated that adding narrative to AVG play stimulates a more active, enjoyable, and whole-body gaming experience than does the same AVG play without a narrative^10,11^. Narrative, the most basic form of communication^12,13^, was found to be a possible additive motivational strategy for increasing physical activity in children^10^, but this has yet to be confirmed in other age populations such as young adults.

Of particular relevance here is whether adding narrative to AVGs or SVGs improves the independent effects or the additive effects of such games on the cognitive function in young adults. Neuroimaging studies have confirmed that the prefrontal cortex (PFC), located at the front of the frontal lobe, appears to be the most important substrate in the control of various cognitive functions, including attention^14^, working memory^15^, inhibitory control^16^, and reasoning^17^. In addition, it has been suggested that the PFC, which forms part of an attentional control in a fronto-parietal network, includes the frontal eye field that modulates processing in the visual cortex with attention^18^ and controls eye movements essential for playing sedentary action video games (e.g., a first-person shooter game)^19^. Narrative^20^, physical exercise^16^, and video game^21^ appear to be potential stimulators of neural activity facilitating cognitive processing in the PFC or the fronto-parietal networks. However, to our knowledge, no studies have examined the independent or additive effects of narrative, physical exercise, or video games through the use of AVGs or SVGs on cognitive function in healthy, young adults.

A narrative process appears to be associated with neural activity in the medial and lateral PFC for selecting and sequencing information of narrative comprehension and production^22,23^, as well as the dorsolateral PFC for the ordering of events within a narrative^24^, which may support cognitive function.

Recent studies have indicated that sedentary action video games (e.g., first or third person shooter games) produce positive effects on various cognitive functions, such as attentional functions^25–27^. The cognitive benefits of action video games are related to the enhanced functional connectivity between the attentional and sensorimotor networks^26^. Further, Bavelier and colleagues^25^ report that action video game players reduced activation in the frontal cortex when attentional load was increased, suggesting action video game players might develop more efficient attentional processes as a result of their gaming activity.

Further, many studies utilizing an acute bout of a traditional, physical activity protocol (e.g., cycling, running) have indicated a favorable effect of the exercise on cognitive function in a wide range of populations^28–32^. Animal and human studies of physical exercise have suggested physiological mechanisms with a number of potential mediators including cerebral blood flow^33^, oxygen oxygenation^34^, and neurotrophic factors^35^, which are tightly coupled with neural activity facilitating cognitive processing^36^, especially in the PFC regions^16^. Since narrative, physical exercise, and video games are known to target the PFC regions, these different stimuli may produce an additive effect on cognitive function.

Of the existing AVG studies^37–40^ utilizing a short- or long- term intervention which involved cognitive function tasks, few have employed an acute bout of AVG protocol among children^41^ or adolescents^42^. Some AVG studies have been conducted to test executive functions, specifically inhibitory control^41^ and cognitive flexibility^42^; however, sustained attention and working memory have been seldom investigated^9^. Sustained attention is the ability to maintain the focus on a stimulus of task-relevant information while consciously attempting to ignore other stimuli over a relatively long period^43^. Working memory, also known as short-term memory, is the capacity to store information for brief periods and to manipulate it during storage through three basic processes involving encoding, maintenance, and retrieval^44^. Together, attention facilitates the rapid integration and transfer of new information to the next stage of working memory in information processing^45^. Thus, sustained attention and working memory are critical elements in human information processing for learning new behavior tasks or skills as well as acquiring new knowledge potentially leading to advantages in social, academic, or occupational functioning in early adulthood^46^.

We used a randomized-controlled trial of narrative and AVG with an active comparator, SVG, in healthy, young adults to examine the hypothesis that narrative and AVG, separate or together, would induce beneficial cognitive effects as measured by sustained attention and working memory, and that the combination would additively prompt more engagement in physical activity. If true, such information would provide insight into feasible and effective strategies for developing a successful physical activity regimen and cognitive training, thereby promoting health behavior.

## Methods

### Participants

Our study was approved by the Institutional Review Board of Northeastern University and all participants signed a written consent form for their participation. The methods were carried out in accordance with approved guidelines and regulations. We recruited through web advertisements and flyers healthy young adults of various ethnic backgrounds who spoke English. Participants were eligible if they met the following conditions: (1) were between 18 and 25 years old; (2) were free of cardiovascular, cerebrovascular, or neurological diseases, attentional disorders, or physical disability; (3) were not a current or former user of tobacco; and (4) had never previously played the video games used in our study. We reviewed the information provided by participants via online prescreening questions to select qualified eligible participants. We screened 853 individuals, of whom 148 were eligible; of these 148, 38 failed to attend their scheduled study sessions, leaving 110 eligible participants. Using a list of computer-generated random numbers, we then randomly assigned the remaining 110 participants to one of four experimental groups: (a) AVG with narrative (N-AVG); (b) AVG without narrative (AVG); (c) SVG with narrative (N-SVG); and (d) SVG without narrative (SVG). Of the 110, 5 had incomplete cognitive data and 5 did not have physical activity data collected due to a technical error, leaving 100 eligible participants for data analysis and an equal number of participants in each group (n = 25) as shown in Table 1.

**Table 1:** Participants’ characteristics.

Variable	Total	N-AVG	AVG	N-SVG	SVG	Statistics	
	Mean ± SD	Mean ± SD	Mean ± SD	Mean ± SD	Mean ± SD	*F*	*p*
Age, years	21.33 ± 2.16	21.28 ± 2.23	21.28 ± 2.35	21.32 ± 2.15	21.44 ± 2.02	0.03	0.993
Male/Female, n	55/45	14/11	13/12	14/11	14/11		
Race, %							
African American	6.00	4.00	—	8.00	12.00		
Asian	42.00	40.00	40.00	40.00	48.00		
Caucasian	36.00	40.00	40.00	32.00	32.00		
Hispanic	10.00	12.00	12.00	8.00	8.00		
Other	6.00	4.00	8.00	12.00	—		
Years of Education	3.27 ± 1.36	3.20 ± 1.47	3.24 ± 1.26	3.24 ± 1.33	3.40 ± 1.44	0.10	0.958
Height, cm	171.75 ± 10.09	172.73 ± 11.17	172.25 ± 10.28	171.28 ± 9.84	170.76 ± 9.51	0.19	0.901
Weight, kg	69.07 ± 14.31	66.42 ± 11.34	70.24 ± 19.17	70.92 ± 14.08	68.73 ± 11.75	0.48	0.698
Body mass index, kg/m^2^	23.33 ± 3.93	22.12 ± 2.15	23.66 ± 5.94	24.05 ± 3.43	23.49 ± 3.17	1.15	0.333
Hours Slept Prior to Study	7.11 ± 1.28	6.70 ± 1.17	7.02 ± 1.15	7.40 ± 1.47	7.30 ± 1.27	1.54	0.209
Weekly Physical Activity, MET	3625.18 ± 2093.38	3302.48 ± 2167.34	3764.80 ± 1692.63	3769.76 ± 1724.53	3663.68 ± 2718.15	0.27	0.846
Daily Sitting Time, min	450.00 ± 168.57	439.20 ± 146.68	493.20 ± 207.32	427.20 ± 158.81	440.00 ± 157.49	0.76	0.522

MET, estimated metabolic equivalent; N, narrative; AVG, active video game; SVG, sedentary video game.

### General procedure

We estimated that, during their one study visit, each participant would spend approximately 90 minutes in the lab. We asked participants to refrain from engaging in strenuous exercise and consuming alcoholic, caffeinated or other beverages other than water for at least 24 hours before the study. Once they arrived at the lab, all participants signed an informed consent document explaining the study procedures and the potential risks. After providing the participants with an orientation regarding our study procedures, we asked them to complete a study questionnaire on their video gaming experience and hours of sleep the night before. Participants also completed the International Physical Activity Questionnaire for adults, a self-reported, 7-day recall questionnaire that assesses the frequency, duration, and intensity of physical activity^47^. We calculated their physical activity score following the standard approach as the sum of the products of total time in each of the three categories and a metabolic equivalent value (3.0 for light, 4.0 for moderate, and 7.5 for vigorous physical activity) assigned to each category^48^. Trained research assistants measured stature (to 0.1 cm) using the ShorrBoard (Weight and Measure, LLC, Olney, MD, USA) and body mass (to 0.1 kg) using the SECA scale (SECA, Chino, CA USA) and computed body mass index (kg/m^2^). The participants were then assigned to groups, instrumented devices, and administrated the group protocol as described below.

### Cognitive assessment and group protocol

After participants relinquished their watches and cell phones, we placed devices to measure physical activity and heart rate and then administered a pre-experimental cognitive test using a computerized cognitive assessment of sustained attention and working memory. The device placement and cognitive test procedure are described below. Research assistants then provided instruction for playing a 30-min bout of the assigned condition. Participants played in a private room while the research assistant monitored the play time.

**Figure 1:**
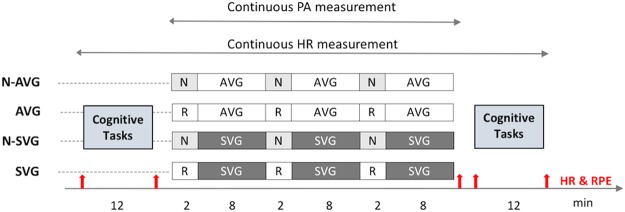
Experimental design and protocol sequences. N, narrative; R, passive rest; AVG, active video game, SVG, sedentary video game; HR, heart rate; RPE, rating of perceived exertion; PA, physical activity.

Figure 1 shows the 30-min experimental conditions for the four groups. In the N-AVG and AVG, participants played for a total of 30 minutes consisting of three 10-minute play intervals comprising 2 minutes of watching narratives (a story mode) or 2 minutes of passive rest, respectively, and 8 minutes of physically playing *Kung-Fu Kinect* with an Xbox One console (Microsoft Inc., Medway, MA USA); the latter was a physical fighting video game in which the Kinect sensor captured (hands free) full-body 3D motion, voice and facial recognition. While playing the game, a participant could see his/her own body in the screen and fought enemies by punching, kicking, or jumping.

In the N-SVG and SVG groups, participants played for the same time periods (including intervals of identical length for narrative and passive rest) but played the SVG *Street Fighter V* using the PlayStation 4 console (Sony Interactive Entertainment, LLC, San Mateo, CA, USA). This was a sedentary, fighting video game using a hand-held controller including gameplay based on eight directions of movement and six attack buttons with a 2D series fighting. Participants selected fighters and then played against their opponents using a variety of attacks and special abilities. This SVG was used as an active comparator to the AVG with the same experimental procedure since both AVG and SVG had a similar video gaming content of fighting and combat.

**Figure 2:**
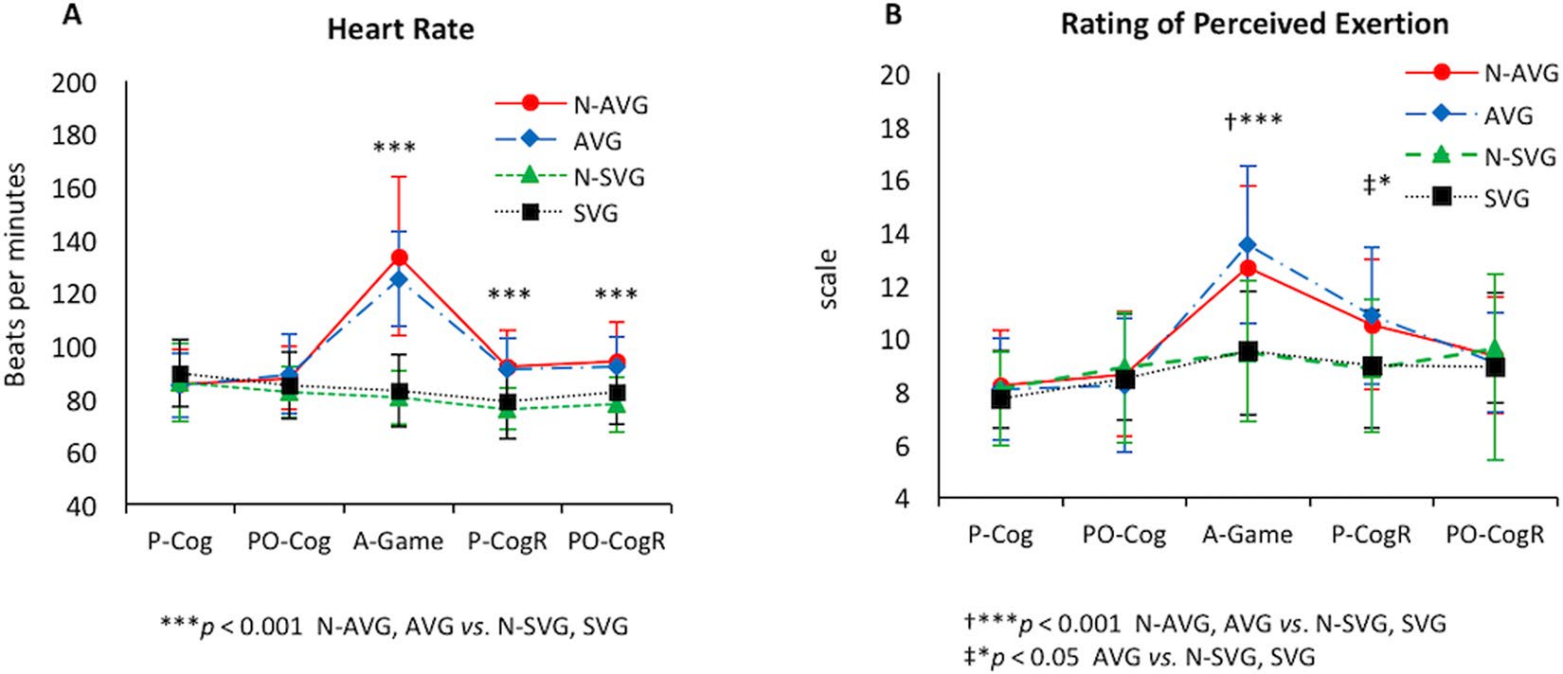
Change in heart rate and rating of perceived exertion during the whole experimental period. N, narrative; AVG, active video game; SVG, sedentary video game; P-Cog, pre-cognitive test; PO-Cog, post-cognitive test/before video game; A-game, immediately after 30-min bout of the assigned game; P-CogR, pre-cognitive re-test; PO-CogR, post-cognitive re-test. Data are presented as means ± standard deviation.

We administered a post-experimental cognitive test after the 30-min bout of the assigned experimental condition when their heart rate returned to 10% above their baseline heart rate (especially in the N-AVG and AVG groups). We also recorded their heart rate from an LCD screen displaying real-time heart rate in the wrist-worn GT9X accelerometer (Fig. 2A) and collected the Borg rating of perceived exertion^49^ over the five time-points (Fig. 2B): (1) pre-cognitive test, (2) post-cognitive test/before video game; (3) immediately following 30-min bout of the assigned experimental condition, (4) pre-cognitive re-test; and (5) post-cognitive re-test. In addition, the heart rate collected at the (2) post-cognitive test/before video game was used as baseline hear rate since participants had a seated rest for 20 minutes prior to the experimental condition.

### Device placement and accelerometry analysis

For participants in all four groups, we placed a Polar H7 Bluetooth heart rate sensor (Polar Electro Inc., Lake Success, NY, USA) on the chest with a soft textile strap and ActiGraph tri-axial accelerometer (GT9X; ActiGraph LLC, Pensacola, FL) with a silicone band on the non-dominant wrist to measure heart rate and upper body movements, respectively. We later fitted participants in both AVG groups with another ActiGraph GT3X+ with a belt clip at the anterior axillary line of the non-dominant hip to measure lower body movements. Both GT9X and GT3X+ accelerometers had an excellent inter-instrument reliability on both the hip-worn accelerometers (Intra-class correlation: ICC = 0.93, *p* < 0.001) and the wrist-worn accelerometers (ICC = 0.89, *p* < 0.001).

**Figure 3:**
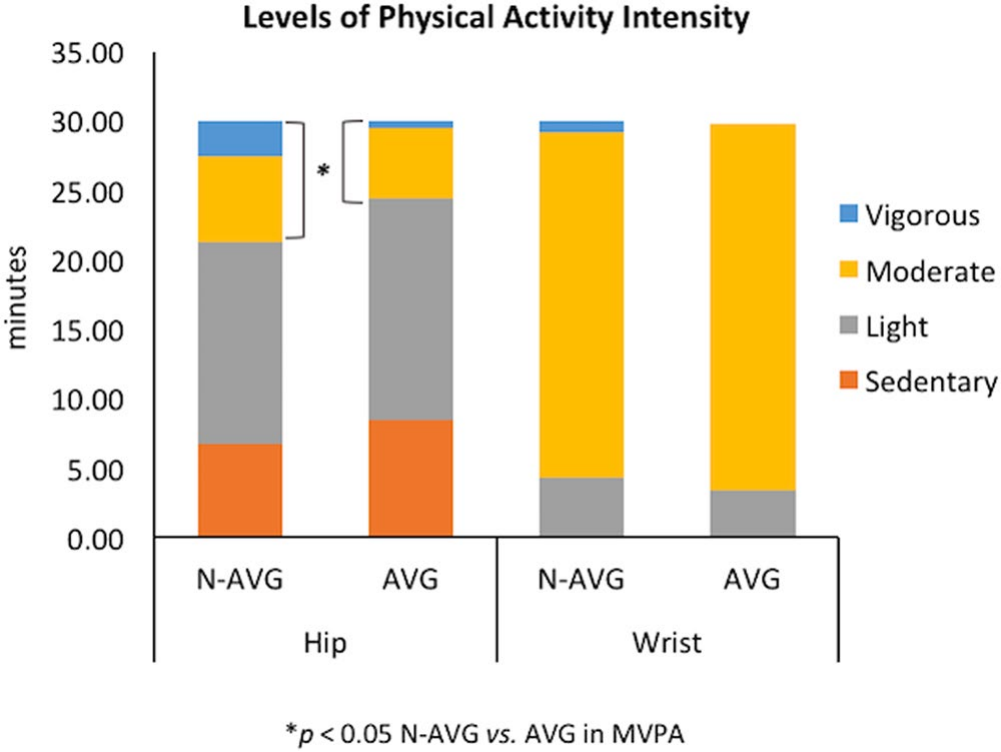
Average time spent on physical activity intensity. N, narrative; AVG, active video game. Data are presented as means (minutes).

For accelerometry processing and analysis, the ActiGraph tri-axial accelerometers measure accelerations from the subject’s intensity and frequency of movement in three individual axes (anterior-posterior, vertical, medial-lateral) and were initialized at 30 Hz sampling (i.e., collect acceleration data 30 times per second in each axis). We used ActiLife software v.6.13.2 (ActiGraph LLC, Pensacola, FL) to download these data from the activity monitors and to convert acceleration data into the three axes activity counts, which quantify the amplitude and frequency of detected accelerations at a 10-sec epoch dataset (i.e., a user-specified time interval). The sum of the activity counts in counts per minutes is associated with activity intensity and can be categorized based on validated activity count cut-points (e.g., a 60-sec epoch: sedentary, ≤100; light, 101–2019; moderate, 2020–5998; vigorous, ≥5998)^50^. The length of time in each activity is as expressed in minutes (Fig. 3). We later combined moderate and vigorous physical activity into a moderate-to-vigorous physical activity category for data analysis.

**Figure 4:**
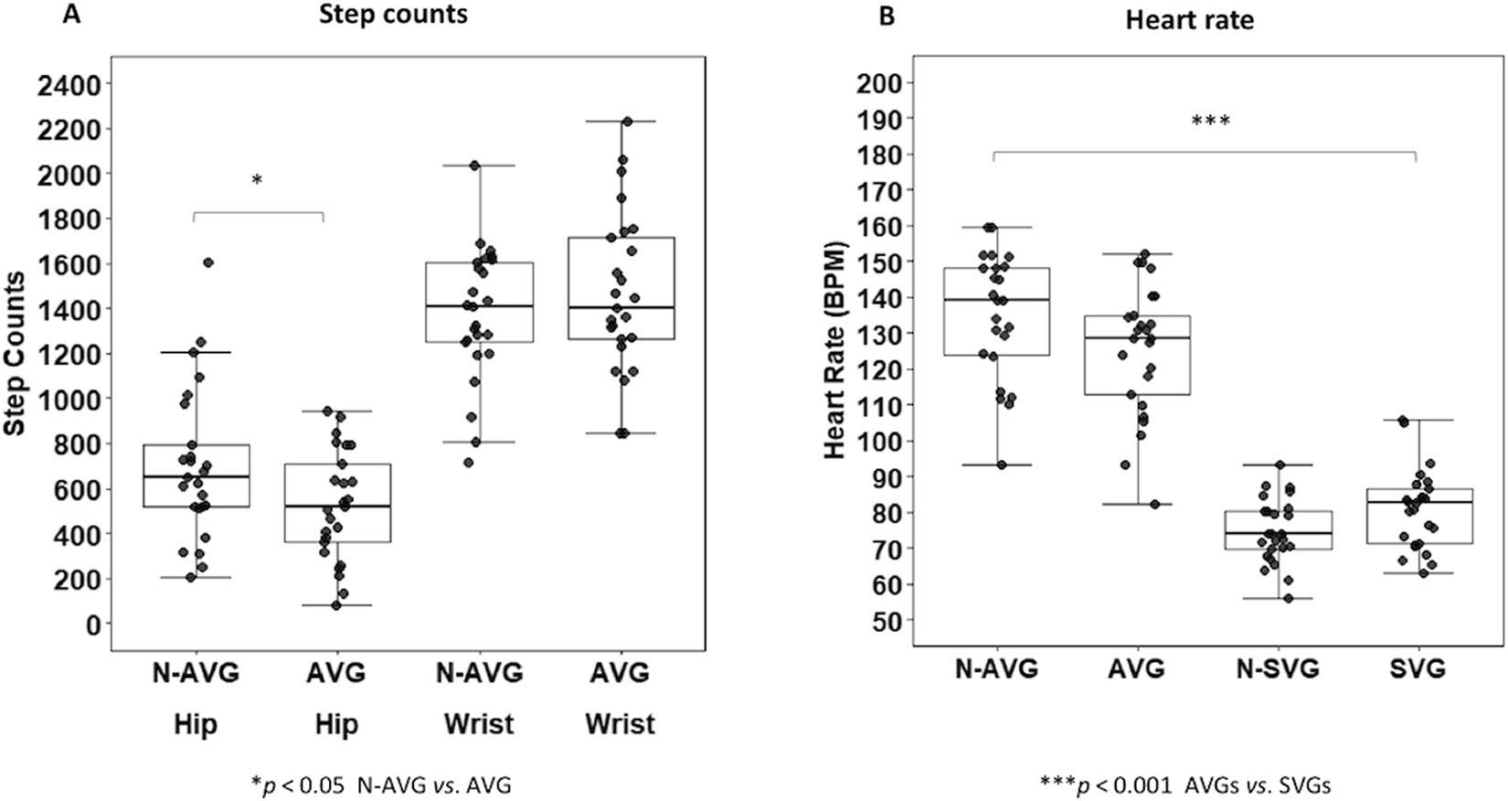
Average step counts and heart rate during the 30-min experimental condition. N, narrative; AVG, active video game; SVG, sedentary video game; BPM, beats per minutes. Box plot with scatter represents 25^th^ percentile at bottom and 75^th^ percentile at top with the highest, median, and lowest value.

We defined step counts as the number of steps the subject took, which was calculated from the built-in algorithm of the ActiLife software using a zero-crossing method based on raw accelerations from the vertical axis^51^. Further, we set the Bluetooth wireless function for a wrist-worn GT9X accelerometer in the ActiLife software to integrate with a Polar H7 Bluetooth heart rate sensor worn in proximity to the heart to measure the subject’s heart rate in beats per minute. We also used electrode conductivity gel for better signal conduction between the skin and the chest transmitter electrode. Since the monitors automatically collected and stored data of step counts and heart rate, we also downloaded a 10 s-epoch dataset of step counts from a 30-min bout of AVG (Fig. 4A) and heart rate from a 30-min bout of AVG or SVG play (Fig. 4B). We summed up the step counts and averaged the heart rate for data analysis.

### Cognitive procedure and analysis

Participants underwent two cognitive tasks in random order. We provided the participants with instructions on how to perform the tasks and a one-min practice trial of each cognitive task before they commenced the cognitive assessment.

**Figure 5:**
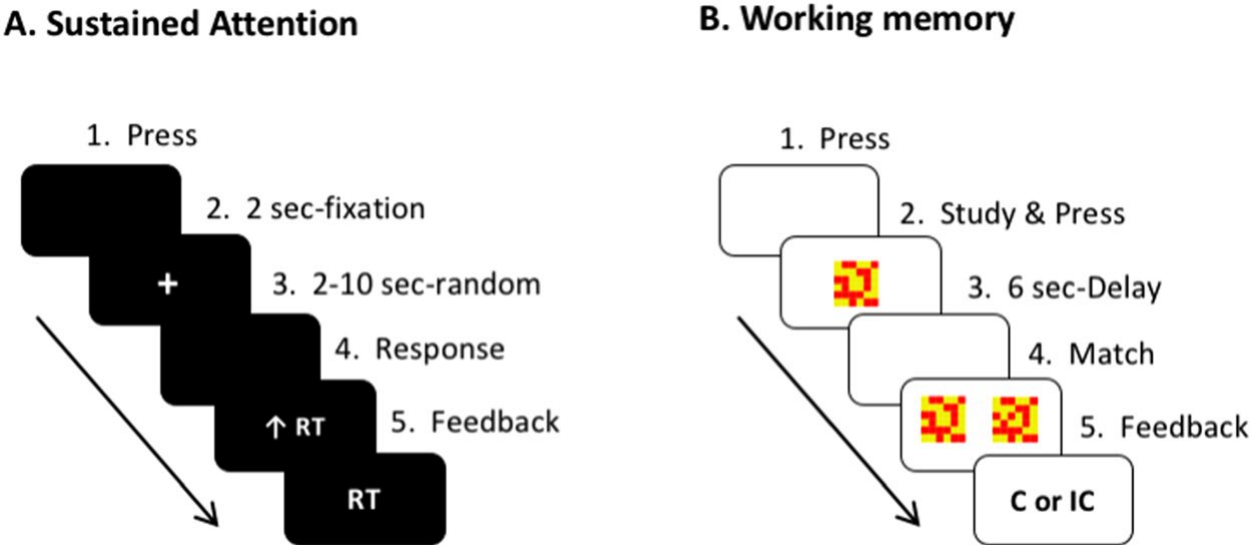
Sustained attention and working memory tasks. Sustained attention as depicted in (**A**) was tested through a psychomotor vigilance task providing feedback for reaction time (RT, msec). Working memory as illustrated in (**B**) was assessed through a delayed match-to-sample memory task providing feedback for either “correct (C)” or “incorrect (IC)” trial.

We assessed sustained attention using a computer-based psychomotor vigilance task (Fig. 5A)^52^ mediated by frontal cortical regions^53^ and posterior parietal cortex^54^. Participants performed four blocks of trials, each block containing 10 trials. In each trial, participants were instructed to attend to a small, fixed point at the center of a computer screen for 2 seconds (sec) and then respond via button press as rapidly as possible between 100 milliseconds (msec) and 500 msec upon detection of a msec-counter on the screen. The stimulus was presented at random time intervals between 2 and 10 sec. The final counter values corresponded to the participant’s reaction time. Response success (within a range of 100–500 msec) or failure (“gun-jump” <100 msec or “lapses” >500 msec) was displayed on the window screen for 1 sec, thus providing feedback for reaction time (msec), and the participant’s data were stored on the computer as the average reaction time (msec) and the number of correct trials and errors. The task took approximately 5 minutes^55^.

We assessed working memory using a computer-based delayed match-to-sample memory task (Fig. 5B) mediated by the prefrontal cortex^56^, the posterior orbitofrontal cortex^57^, and the hippocampus^58^. Participants performed three blocks of trials, each block containing 10 trials. With an initial key press in each trial, participants viewed and studied a 6 × 6 grid of brightly colored yellow and red squares with a unique pattern on a computer screen. After another key press, the stimulus disappeared and the screen became blank for a 6 sec-delay period, and then two stimuli were presented on the screen (a “match” and “non-match”). Participants were asked to indicate which stimulus was the correct “match” with a key press and to respond as quickly and as accurately as possible, thus providing feedback for either “correct” or “incorrect” trial in 1 sec. After completing 30 trials for the average 7.68 min, the final counter values were stored on the computer as the average encoding (msec), retrieval latency (msec), and the number of correct trials (score). The encoding time and retrieval latency depended on how long a participant viewed each pre-delay stimulus, how long a participant examined each post-delay stimulus and whether the participant chose the “match,” respectively. The number of correct trials indicated response accuracy^55^.

We implemented the psychomotor vigilance task and the delayed match-to-sample memory task using a program called the Psychology Experiment Building Language (PEBL, an open-source programming language)^59^ run on a Windows computer in a closed office. The data gathered by the PEBL program produced output in text file format, including each trial’s cognitive processing time in msec and a number indicating whether the trial was a success or a failure^55^.

### Statistical analysis

We examined all continuous variables for linearity using scatterplots and for normality via visual inspection of a histogram and Q-Q plot with skewness and kurtosis. We observed a linear relationship between variables. Data were normally distributed as indicated by the mean values for skewness (0.473) and kurtosis (0.919), which were within the acceptable range between −1.96 and +1.96^60^.

We used a two-way repeated measure analysis of variance (ANOVA) to: (1) assess the changes in HR and RPE across the five time-points in the four groups; (2) compare the counts of steps taken during the AVG play between the N-AVG group and the AVG group; (3) evaluate levels of physical activity intensity (sedentary, light, moderate, vs. vigorous) and moderate-to-vigorous physical activity between the N-AVG group and the AVG group; and, (4) compare the mean of the heart rate in all four groups.

For cognitive data analysis, we performed a two-way repeated measures ANOVA using a 2 × 4 design to evaluate the effect of the two time-points (pre *vs*. post) and the four group experimental conditions (N-AVG, AVG, N-SVG, *vs*. SVG) on cognitive outcomes. We conducted a three-way repeated measures ANOVA using a 2 × 2 × 2 design to check on the influences of the predominant factor (AVG or the narrative) on cognitive outcomes: (1) the two time-points (pre *vs*. post); (2) the video game conditions (AVG: N-AVG *plus* AVG *vs*. SVG: N-SVG *plus* SVG); and (3) the narrative conditions (narrative: N- AVG *plus* N-SVG *vs*. non-narrative: AVG *plus* SVG). When we observed an interaction or main effect, we performed a post hoc multiple comparison test to identify differences in the mean of step count, heart rate, and levels of physical activity and in the mean of pre-post change in cognitive outcome.

We calculated the Pearson’s correlation coefficient to evaluate the relationship between the mean heart rate during AVG or SVG play and the change in pre-post measures of cognitive outcome. All statistical data analyses were conducted with SPSS 23.0 (SPSS Inc., Chicago, IL). The criterion for significance was *p* < 0.05 (two-tailed). For a repeated measures ANOVA, we used partial eta-squared (*η*^2^) as a measure of effect size for significant results. *η*^2^ classified effect sizes as small (0.01), medium (0.06), or large (0.14)^61^.

## Results

### Descriptive results

As shown in Table 1, the 100 participants were, on average, in their early 20 s (21.30 ± 2.14 years), received more than 3.33 years of formal education beyond high school; our study population was 55% male and 45% female, and consisted of 6% African American, 42% Asian, 36% Caucasian, 11% Hispanic, and 6% other ethnicity. The ratios of sex (*X*^2^ = 0.12, *p* = 0.989) and of race/ethnicity (*X*^2^ = 7.80, *p* = 0.801) for the experimental groups were well balanced and similarly distributed. The four groups were also not significantly different with respect to baseline characteristics, age, body mass index, years of education, level of physical activity, sedentary behavior, and hours slept prior to study visit (all, *p* > 0.05).

### Physiological and psychological indicators of exercise intensity

Changes in heart rate and rating of perceived exertion across the five time-points among the four groups are presented in Fig. 2A and B. We found a significant time × group interaction (*F*_3,99_ = 13.68, *η*^2^ = 0.30; *p* < 0.001), showing that heart rate in either N-AVG (133.76 ± 29.99) or AVG (125.21 ± 18.26) was increased similarly after AVG play (*p* < 0.001) and then progressively decreased (Fig. 2A). The heart rate in the AVG groups following the AVG play was still significantly higher than either that in the N-SVG group or the SVG group at both pre-test cognitive re-assessment (*p* < 0.001) and post-test cognitive re-assessment (*p* < 0.001). In addition, we found a significant time effect (*F*_3,99_ = 39.52, *η*^2^ = 0.29; *p* < 0.001) but not a time × group interaction effect (*F*_3,99_ = 0.52, *η*^2^ = 0.02; *p* = 0.667) (Fig. 2B), demonstrating that rating of perceived exertion in either the N-AVG group (12.64 ± 3.10) or the AVG group (13.52 ± 2.95) group increased more than that in either the N-SVG group (*p* < 0.001) or the SVG group (*p* < 0.001) following the video game play. The increased rating of perceived exertion in the AVG group (not the N-AVG group) remained until the post-cognitive re-assessment, and was significantly higher than that in N-SVG (*p* = 0.023) or SVG (*p* = 0.037).

### Physical activity, step counts, and hear rate between narrative AVG and non-narrative AVG

As depicted in Fig. 3 and supplementary Table 1, the 30-min experimental period had non-significant effects on levels of physical activity intensity as assessed either via the hip-worn accelerometer (*F*_1,49_ = 3.03, *η*^2^ = 0.06; *p* = 0.088) or the wrist-worn accelerometer (*F*_1,49_ = 0.01, *η*^2^ = 0.00; *p* = 0.942). However, we found a significant physical activity × group interaction (*F*_1,49_ = 4.86, *η*^2^ = 0.10; *p* = 0.032) due to more time spent on moderate-to-vigorous physical activity with the hip-worn accelerometer (*p* = 0.047) in N-AVG (8.76 ± 6.98) than AVG (5.54 ± 3.68); we did not see this effect for the wrist-worn accelerometer (25.74 ± 3.97 *vs*. 26.42 ± 4.19, respectively_,_ *p* = 0.561). Figure 4A shows the step counts assessed at hip and wrist during the 30-min experimental period for N-AVG and AVG. We observed a significant step count × group interaction (*F*_1,49_ = 6.28, *η*^2^ = 0.12; *p* = 0.016); on closer examination, we found that there were higher step counts as assessed using a hip-worn accelerometer (*p* = 0.031) in N-AVG (699.92 ± 337.53) than AVG (524.32 ± 244.03) but this was not with the case for the wrist-worn accelerometer (1373.12 ± 297.17 *vs*. 1463.56 ± 356.36_,_ *p* = 0.360). Further, as shown in Fig. 4B, the average heart rate during the 30 min-experimental condition was significantly different (*F*_3,99_ = 90.46, *η*^2^ = 0.74; *p* < 0.001) between the AVG groups [N-AVG (132.64 ± 21.17) or AVG (125.41 ± 18.17)] and the SVG groups [N-SVG (74.68 ± 9.06) or SVG (80.83 ± 11.14)]. However, we found no significant difference between N-AVG and AVG (*p* = 0.107) or between N-SVG and SVG (*p* = 0.169).

### Working memory and sustained attention among four groups

**Figure 6:**
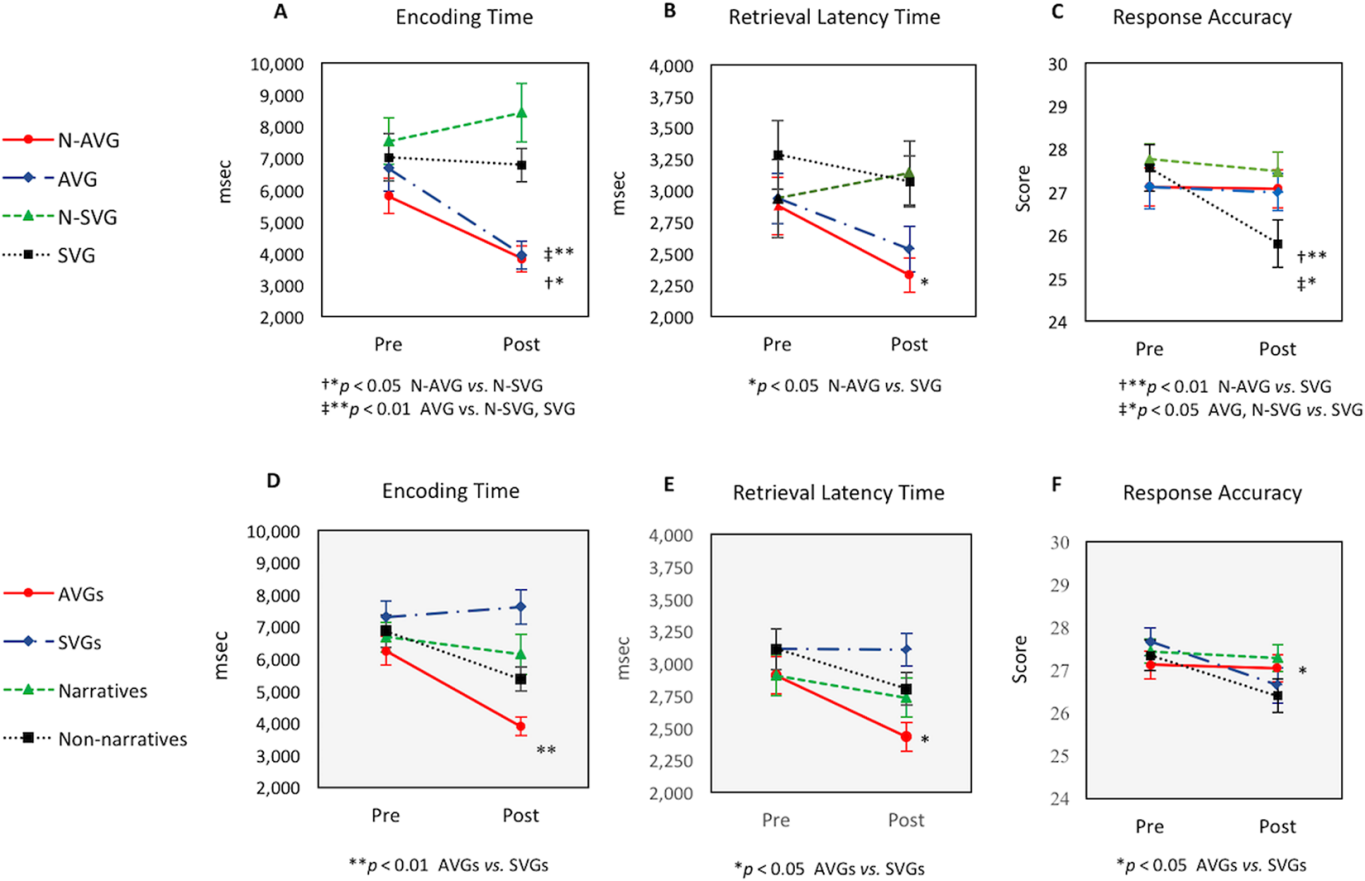
Working memory performance in Delayed Match-to-Sample Memory Task. N, narrative; AVG, active video game; SVG, sedentary video game; AVGs, N-AVG *plus* AVG; SVGs, N-SVG *plus* SVG. Narratives, N-AVG *plus* N-SVG; Non-Narratives, AVG *plus* SVG. Data (means ± standard error) shown in (**A**–**C**) are from a 2 × 4 design of working memory while those represented in (**D**–**F**) are from a 2 × 2 × 2 design of working memory.

The pre-post measures of change in encoding time, retrieval latency time, and correct trial in the working memory task among the four groups are presented in Fig. 6A–C and supplementary Table 2. Using a 2 × 4 design, we found a significant time × group interaction (*F*_3,99_ = 4.97, *η*^2^ = 0.13; *p* = 0.003) in encoding time, suggesting that an encoding time was shorter in N-AVG than N-SVG (*p* = 0.007) and also shorter in AVG than N-SVG (*p* = 0.001) and SVG (*p* = 0.019) (Fig. 6A). Further, we found a significant time effect of retrieval latency time (*F*_3,99_ = 4.37, *η*^2^ = 0.04; *p* = 0.039), suggesting that the four groups’ retrieval latency period decreased over time, but N-AVG had a shorter retrieval latency time compared to SVG (*p* = 0.025) (Fig. 6B). For the response accuracy, we found a significant time × group interaction in correct trial (*F*_3,99_ = 3.23, *η*^2^ = 0.09; *p* = 0.026), indicating that response accuracy in N-AVG (*p* = 0.008), AVG (*p* = 0.012), and N-SVG (*p* = 0.023) was improved compared to SVG (Fig. 6C). However, we found no significant effects of time and group or significant interaction effects on pre-post measures of change in response accuracy, gun jumps, lapse, and reaction time in the sustained attention task between the four groups, suggesting that N-AVG, AVG, N-SVG, and SVG had similar sustained attention performances without any additive effect from narrative (Table 2).

**Table 2:** Sustained attention performance in Psychomotor Vigilance Task.

Variable	N-AVG		AVG		N-SVG		SVG		Statistics	
	Mean ± SD		Mean ± SD		Mean ± SD		Mean ± SD		*Time* × *Group*	
	Pre	Post	Pre	Post	Pre	Post	Pre	Post	*F*	*P*
Reaction Time, msec	371.13 ± 33.71	377.25 ± 26.74	357.90 ± 26.76	364.25 ± 29.69	358.74 ± 30.29	361.72 ± 27.71	361.33 ± 32.62	364.33 ± 31.54	0.21	0.893
Gun jumps, score	1.00 ± 1.55	1.16 ± 1.31	1.84 ± 2.19	1.56 ± 1.46	1.64 ± 1.38	2.08 ± 2.14	1.68 ± 1.95	1.92 ± 1.22	0.59	0.626
Lapse, score	1.40 ± 1.85	2.16 ± 2.19	1.28 ± 2.20	2.48 ± 4.22	2.08 ± 3.37	2.12 ± 3.02	2.24 ± 2.70	2.68 ± 4.11	0.78	0.508
Correct trials, score	37.6 ± 2.42	36.68 ± 2.54	36.88 ± 2.81	35.96 ± 4.78	36.28 ± 3.95	35.80 ± 4.21	36.08 ± 3.95	35.40 ± 4.39	0.10	0.960

N, narrative; AVG, active video game; SVG, sedentary video game.

### Working memory between AVG groups and narrative groups

We used a 2 × 2 × 2 ANOVA to see whether narrative or AVG had an effect on the improvement of working memory performance (Fig. 6D–F and supplementary Table 3). We found that the AVG groups had a shorter encoding time than did the SVG groups, as demonstrated by a time × AVG interaction (*F*_1,99_ = 13.21, *η*^2^ = 0.12; *p* < 0.001), but we did not find a significant narrative effect (*F*_1,99_ = 1.64, *η*^2^ = 0.02; *p* = 0.203) or narrative × AVG effect on the encoding time (*F*_1,99_ = 0.07, *η*^2^ = 0.01; *p* = 0.794) (Fig. 6D). Further, the retrieval latency time was improved as indicated by a time × AVG interaction (*F*_1,99_ = 4.17, *η*^2^ = 0.04; *p* = 0.044) but this effect was not produced by the addition of the narrative (*F*_1,99_ = 0.33 *η*^2^ = 0.01; *p* = 0.565) (Fig. 6E). In addition, we found a significant time × AVG interaction (*F*_1,99_ = 4.32, *η*^2^ = 0.04; *p* = 0.040), indicating that the AVG groups had an enhanced response accuracy compared to the SVG groups, whereas we found no significant effects for either the time × narrative interaction (*F*_1,99_ = 2.97, *η*^2^ = 0.03; *p* = 0.088) or the time × AVG × narrative interaction (*F*_1,99_ = 2.39, *η*^2^ = 0.02; *p* = 0.125) (Fig. 6F).

### Correlation analyses between heart rate and change in cognitive variables

**Figure 7:**
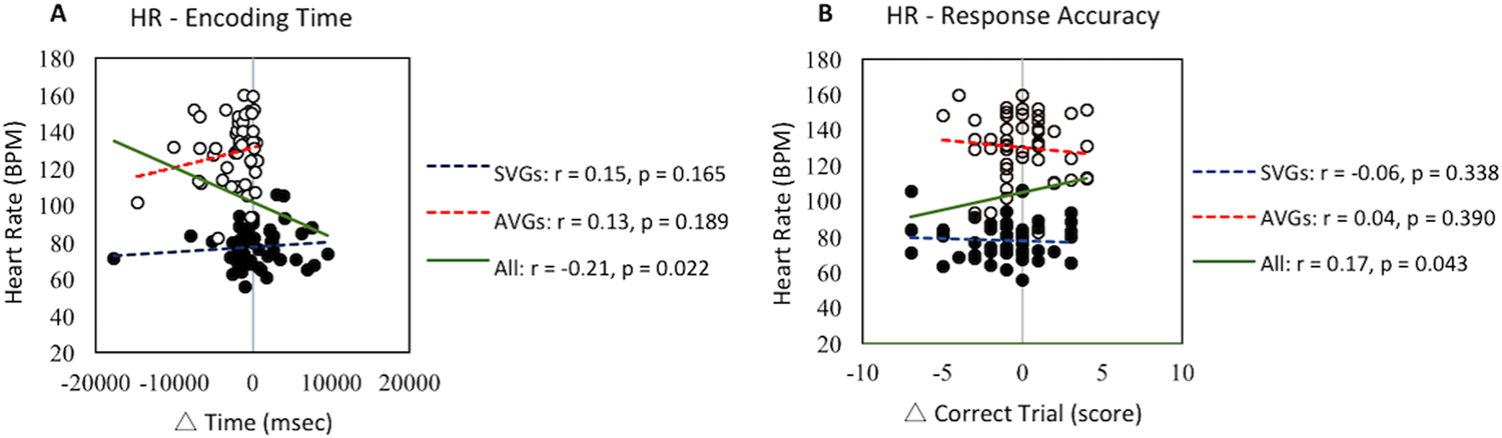
Relationship between average heart rate during the 30-min experimental condition and pre-post change of working memory. BPM, beats per minutes. White circles depict AVGs (active video games: N-AVG *plus* AVG) participants while black circles represent SVGs (sedentary video games: N-SVG *plus* SVG) participants. The red, blue, and green dotted lines represent linear regression for AVG participants, SVG participants, and all participants, respectively.

The average heart rate during the 30-min experimental period was not significantly correlated with the pre-post change in encoding time (Fig. 7A) or response accuracy (Fig. 7B) of working memory task in either the AVG groups (*r* = 0.13, *p* = 0.189 and *r* = 0.04, *p* = 0.390, respectively) or the SVG groups (*r* = 0.15, *p* = 0.165 and *r* = −0.06, *p* = 0.338, respectively). In all participants, however, the average heart rate was significantly correlated with the pre-post change of shorter encoding time (*r* = −0.21, *p* = 0.022), as depicted in Fig. 7A, and higher response accuracy (*r* = 0.17, *p* = 0.043), as shown in Fig. 7B. The average heart rate, though, was not related to retrieval latency time in either the AVG groups (*r* = 0.19, *p* = 0.095), the SVG groups (*r* = 0.20, *p* = 0.089) or all participants (*r* = −0.04, *p* = 0.337).

## Discussion

To our knowledge, this is the first randomized-controlled study demonstrating that adding narrative to AVG additively increases physical activity and enhances cognitive function in healthy, young adults. The non-significant group differences we observed suggest that our attempts to control known confounding factors were successful, particularly those we explicitly addressed (e.g., sex, physical activity, obesity indices, education years, hours slept)^30,55,62,63^. Adding a narrative as a motivational tool and a cognitive component to an AVG increased participants’ physical activity level, but did not additively improve cognitive function. While both the AVG and SVG groups achieved continued maximal performance in sustained attention, only the AVG group that induced moderate-intensity exercise was the primary factor for overall working memory enhancement.

We found that the 30-min AVG intervention in the N-AVG and AVG groups resulted in a moderate-intensity level of physical activity, corresponding to the recommended 30-min of moderate-intensity physical activity per day for adults. For instance, using the average heart rate and the rating of perceived exertion we measured during and immediately after the 30-min AVG-induced exercise, respectively, as physiological and psychological indicators of exercise intensity, our participants achieved a moderate-intensity level of physical activity^64^ in both the narrative-AVG and non-narrative-AVG. We found that adding a narrative to an AVG yielded 23% more steps than AVG alone. Our results are similar to those of Lu and colleagues^10^ that demonstrated that watching a narrative cutscene before AVG play of similar duration with a Nintendo Wii increased physical activity level by 40% more steps (compared to a non-narrative group) in overweight and obese children. Since step counts represent the amount of physical activity during AVG play, we further analyzed the levels of physical activity intensity from accelerometer monitors and showed that players in the narrative-AVG group had 11% more moderate to vigorous physical activity level than did those in the AVG-only group. Confirming findings from previous work in children^10^, our young adults were more physically active at higher intensity levels of physical activity when a narrative was added to AVG. Thus, the addition of a narrative to AVGs could promote strong intrinsic motivation by inclining attention to the game, promoting instant enjoyment for a new type of game, triggering exploration of the game environment^65^, and absorbing players in an immersive fictional world^66^, thereby stimulating a more physically-active movement^10^. Falzon and colleagues^67^ using a survey instrument showed that a narrative might be an effective delivery method for improving intention and positive beliefs regarding physical activity in sedentary, middle-aged women with breast cancer undergoing chemotherapy. Taken together, our results and those from other studies suggest that a narrative is an effective motivational strategy for increasing physical activity and promoting physical activity behavior, regardless of age and health status.

An acute bout of traditional physical exercise at moderate-to-vigorous intensity has been employed by previous researchers to provide insight into the discrepant findings reported in the physical activity-cognitive intervention literature^28–32,68^. In line with the traditional physical exercise studies, we found that participants in our AVG groups performed better on working memory performance as indicated by more efficient encoding times, more efficient retrieval latency times, and higher response accuracy of information than did those in our SVG groups. Narrative did not contribute significantly additively to AVG or SVG; however, adding a narrative to SVG improved response accuracy in part of the working memory task compared to SVG alone. Participants in the AVG groups also had a better overall working memory performance than those in the SVG groups, although there was no narrative effect on any cognitive function. These findings suggest that the physical exercise obtained from playing AVG was the dominant component-related aspect of cognitive enhancement, especially in working memory. Our findings are consistent with a recent meta-analysis study^9^ which indicated that short or long-term AVG interventions improved global cognition along with executive functions, attentional processing, and visuospatial skills in older adults enrolled in clinical or non-clinical trials. Further, two recent studies among young people showed that acute bouts of AVG intervention immediately enhanced cognitive performance on measures of inhibitory control in children^41^ and cognitive flexibility in adolescents^42^. Taken together, physical exercise through AVG appears to be a prime factor for enhancing cognitive function regardless of age group. Thus, our finding from young adults contributes to the literature on AVG and cognition research.

In all four of our experimental groups, we found a non-significant effect related to the pre-post measures of response accuracy, reaction time, and errors in the sustained attention task. Although there was no significant difference between our four groups, we theorize that, regardless of narrative effect, SVG groups and AVG groups similarly maintained sustained attentional performance at almost the maximal levels based on their times and minimal errors in the pre-post measures. A recent study^30^ using the same task showed that only a control group (passive rest) had worse performance as indicated by a longer reaction time, compared to the other treatment groups (i.e., aerobic exercise, transcranial laser stimulation). Since we had no control group but included groups who engaged in AVG and SVG, increased attentional capacity from AVG or SVG might prevent the cognitive fatigue that produces worse performance in the pre-post measure of sustained attention. These findings are in line with other studies among healthy, young adults demonstrating that aerobic exercise improved attention capacities^30,69^ and sedentary action video games enhanced attention abilities^25,70,71^ including visual selective attention^25,70^, spatial distribution of attention, and temporal resolution of attention^71^. Otherwise, we cannot rule out the possibility that our study population of young adults attained the maximal performance in sustained attention due to cognitively-high functioning in terms of age and health status^55^. The psychomotor vigilance task has been widely used among populations with health conditions such as sleep deprivation^62,72,73^ or psychiatric disorders such as ADHD^74^, showing significant dysfunction in sustained attention compared to their counterparts.

The improvement we found in working memory produced by the use of AVG might involve both vigilant and physiological alterations. A recent study^9^ using meta-analysis compared effect size on overall cognition between AVG interventions and regular exercise interventions administering equal amounts of physical activity to determine whether the cognitive benefits of AVG extend beyond regular exercise. The researchers showed moderately greater effects on cognition from AVG compared with regular exercise, demonstrating that AVG outperformed regular exercise. The empirical evidence is limited regarding more cognitive benefits from AVG in comparison to regular exercise. However, Benzing and colleagues^42^ suggested the possibility that better cognitive function in AVG than regular exercise (e.g., running) might be related to a different magnitude of cognitive engagement from the video game play. Brain imaging studies using a sedentary action video game indirectly provide evidence that the video game elevates vigilant status, which is associated with more efficient attentional demands in a fronto-parietal network of areas^25^ and enhanced functional connectivity between the attentional and sensorimotor networks^26^. These might occur during AVG play requiring more attentional ability to imitate and learn new sequences of movements, comparable to regular exercise (e.g., running)^42^. Thus, enhanced attentional capacity during AVG play might support facilitating and transferring the information to working memory in information processing^45^.

The physiological mechanisms by which AVG improves cognition in healthy, young adults are currently unclear. We did not find a significant correlation between the average heart rate during AVG or SVG and any sustained attention or working memory performance, but average heart rate was positively correlated with the pre-post difference of information encoding time and response accuracy in working memory. This suggests that there are physiological mechanisms likely involved in the beneficial effect of physical exercise via AVG on working memory. Physical exercise elicits heart rate and arousal, consequently increasing cerebral blood flow to meet the compensatory metabolic demands (e.g., oxygen, lactate, glucose) of cerebral metabolism for supporting neuronal activity^75^ in regions of the brain during intense cognitive activity^76^. Endo and colleagues^32^ demonstrated that a moderate-intensity physical exercise increased prefrontal oxygenation, which appears to be related to a region in the brain involved with working memory. Brain-derived neurotrophic factor (BDNF), which enhances neuroplasticity by stimulating synaptic connections between neurons during cognitive activity, has emerged as a crucial mediator of the association between physical exercise and cognition^77,78^. An increased serum BDNF immediately following an acute bout of moderate-to-vigorous exercise has been reported to be positively correlated with prefrontal-based cognitive enhancement^29^. Physical exercise-induced physiological alterations might thus be involved in at least part of modulating neural activity, possibly leading to improvements in cognitive function.

We should note some important limitations to our results. Our study population consisted of young adults and may not represent other populations. We had no survey questions that asked participants about their experience with action video game (e.g., a first or third-person shooter video game), which might have influenced their performance^79^. However, we demonstrated that there was no significant difference in the video game experiences with PlayStation and Xbox among the four groups (all, *p* > 0.45). In addition, the psychomotor vigilance task and delayed match-to-sample memory task we used are experimentally validated for sensitivity to fatigue and are not affected by aptitude and learning^30^, suggesting that these tasks may have been sensitive to differences in cognitive performance. As we previously noted, the psychomotor vigilance task we used may not have been the best choice for healthy, young adults especially since we did not have a control experimental condition group (i.e., no passive rest group)^55^. Additionally, the time of day may influence performance on the cognitive tasks^80^; however, we didn’t see any significant correlation between the time of cognitive tasks and any cognitive performance in the psychomotor vigilance task or the delayed match-to-sample memory task (all, *p* > 0.15). Finally, we did not have access to brain imaging or biochemistry and were therefore unable to determine the physiological mechanisms mediating the relationship between physical exercise or narrative through AVG and cognitive function.

In conclusion, we found that healthy, young adults who played AVG performed better on computerized, prefrontal-dependent cognitive task assessing working memory, while those who played AVG or SVG achieved persistent maximal performance in sustained attention. Those who experienced the addition of a narrative to AVG were more physically active at a moderate intensity level of physical activity. Our findings pave the way for a new generation of physical activity training programs for promoting healthy behavior, thereby enhancing the brain’s cognitive health. Future studies should be conducted to confirm our results with brain imaging techniques to elucidate the causal relationship of narrative and AVG between neural activity and cognitive function. Further, it would be useful to establish the durability and long-term effects of narrative and AVG on brain structural integrity and function for individuals with cognitive disorders or working memory deficits.

## Electronic supplementary material

Supplemental Tables
