# Supplementary material for: Narrative and active video game in separate and additive effects of physical activity and cognitive function among young adults
Source: Sci Rep. 2018 Jul 20;8:11020. doi: 10.1038/s41598-018-29274-0 (PMC6054679; doi:10.1038/s41598-018-29274-0)
Supplement: Supplementary file 1 — Supplemental Tables [file 41598_2018_29274_MOESM1_ESM.docx]

#### Narrative and active video game in separate and additive effects of physical activity and cognitive function among young adults

Jungyun Hwang^1,*^, Amy Shirong Lu^1^

^1^ Health Technology Lab, College of Arts, Media and Design, Bouvé College of Health Sciences, Northeastern University, Boston, MA 02115, USA

^*^ Corresponding author

Jungyun Hwang, PhD

Northeastern University

212A Lake Hall, 360 Huntington Avenue

Boston, MA 02115

Phone: 617.373.6331

Email: j.hwang@northeastern.edu

Supplement Table 1. Levels of physical activity intensity (in minutes)

|  | Hip | | Wrist | |
| --- | --- | --- | --- | --- |
|  | N-AVG | AVG | N-AVG | AVG |
|  | Mean ± SD | Mean ± SD | Mean ± SD | Mean ± SD |
| Sedentary | 6.76 ± 6.19 | 8.46 ± 6.80 | – | – |
| Light | 14.48 ± 4.21 | 15.99 ± 5.40 | 4.26 ± 3.97 | 3.37 ± 4.05 |
| Moderate | 6.25 ± 3.46 | 5.07 ± 0.45 | 24.96 ± 5.13 | 26.41 ± 4.18 |
| Vigorous | 2.51 ± 5.65 | 0.47 ± 0.59 | 0.79 ± 3.76 | – |

N, narrative; AVG, active video game; SVG, sedentary video game.

Supplement Table 2. Working memory among four groups

|  | N-AVG | |  | AVG | |  | N-SVG | |  | SVG | |  | Statistics | |
| --- | --- | --- | --- | --- | --- | --- | --- | --- | --- | --- | --- | --- | --- | --- |
|  | Mean ± SD | |  | Mean ± SD | |  | Mean ± SD | |  | Mean ± SD | |  | *Time × Group* | |
|  | Pre | Post |  | Pre | Post |  | Pre | Post |  | Pre | Post |  | *F* | *P* |
| Encoding Time | 5816.49 ±2820.82 | 3830.14 ±2052.25 |  | 6683.72  ±3545.88 | 3945.27  ±2161.29 |  | 7546.68  ±3206.72 | 8435.46  ±4643.01 |  | 7035.15  ±3672.84 | 6786.23  ±2573.52 |  | 4.97 | 0.003 |
| Retrieval Latency Time | 2878.85  ±1165.51 | 2330.02  ±693.57 |  | 2937.94  ±979.66 | 2535.08  ±924.16 |  | 2937.46  ±922.36 | 3137.08  ±1273.57 |  | 3284.16  ±1377.46 | 3072.55  ±1008.93 |  | 1.99 | 0.121 |
| Response Accuracy | 27.12  ±2.19 | 27.08  ±2.27 |  | 27.12  ±2.47 | 27.00  ±2.12 |  | 27.76  ±1.79 | 27.48  ±2.20 |  | 27.56  ±2.74 | 25.80  ±3.35 |  | 3.23 | 0.026 |

N, narrative; AVG, active video game; SVG, sedentary video game.

Supplement Table 3. Working memory between AVGs and SVGs and between narratives and non-narratives

|  | AVG groups | |  | SVG groups | | | Narrative groups | |  | Non-Narrative groups | |  | Statistics | | | |
| --- | --- | --- | --- | --- | --- | --- | --- | --- | --- | --- | --- | --- | --- | --- | --- | --- |
|  | Mean ± S.D. | |  | Mean ± S.D. | |  | Mean ± S.D. | |  | Mean ± S.D. | |  | *Time × AVG* | | *Time × Narrative* | |
|  | Pre | Post |  | Pre | Post |  | Pre | Post |  | Pre | Post |  | *F* | *P* | *F* | *P* |
| Encoding Time | 6250.10 ±3201.17 | 3887.70 ±2086.66 |  | 7290.91 ±3422.07 | 7610.84 ±4246.42 |  | 6681.58 ±3114.09 | 6132.80 ±2755.14 |  | 6859.43 ±3577.29 | 5365.74 ±2759.32 |  | 13.21 | < 0.001 | 1.64 | 0.203 |
| Retrieval Latency Time | 2908.39 ±1065.97 | 2432.55 ±815.26 |  | 3110.81 ±1173.32 | 3105.02 ±1137.58 |  | 2908.15 ±1040.62 | 2733.75 ±1093.79 |  | 3111.05 ±1195.82 | 2803.81 ±995.28 |  | 4.17 | 0.044 | 0.33 | 0.565 |
| Response Accuracy | 27.12 ±2.30 | 27.04 ±2.17 |  | 27.66 ±2.29 | 26.64 ±2.93 |  | 27.44 ±2.00 | 27.28 ±2.22 |  | 27.34 ±2.59 | 26.40 ±2.84 |  | 4.31 | 0.040 | 2.97 | 0.088 |

AVG, active video game; SVG, sedentary video game.
